# Racial Differences in the Association Between School Quality and Adult Health: A Nationally Representative Longitudinal Study

**DOI:** 10.1007/s40615-026-03118-4

**Published:** 2026-08-03

**Authors:** Benjamin P. L. Meza, Kevin Hoang, Christopher Biely, Nicholas Jackson, Kathryn M. Leifheit, Elizabeth Barnert, Adam Schickedanz, Hana Khidir, Sai Iyer, George M. Slavich, Alma D. Guerrero, Rebecca N. Dudovitz

**Affiliations:** 1 UCLA Division of General Internal Medicine and Health Services Research, Department of Medicine, David Geffen School of Medicine, University of California, 1100 Glendon Ave, Suite 900, Los Angeles, California 90024, USA; 2 Walnut Creek Medical Center, John Muir Health, Walnut Creek, California, USA; 3 Department of Pediatrics, David Geffen School of Medicine, University of California, Los Angeles, California, USA; 4 Department of Health Policy & Management, UCLA Fielding School of Public Health, University of California, Los Angeles, California, USA; 5 Department of Medicine, Olive View Medical Center-University of California, Los Angeles, Sylmar, California, USA; 6 Department of Psychiatry and Human Behavior, University of California, Irvine, CA, USA

**Keywords:** School Quality, Health Equity, Racial Disparities, Adolescent Development, Mental Health, Longitudinal Study

## Abstract

Although school quality is associated with greater educational attainment and better health in population-wide studies, it remains unclear whether these effects are experienced similarly across racial and ethnic groups in the USA. To explore this question, we used the nationally representative National Longitudinal Study of Adolescent to Adult Health to examine the association between four individual school quality indicators (student promotion rates, teacher retention, average daily attendance, parental involvement) in Wave I (grades 7–12th, age 12–19), with adult health outcomes in Wave V (age 32–42), stratified by race/ethnicity (White, Black, Latinx). The primary outcomes were self-rated health and depressive symptoms. Regression models adjusted for baseline health, individual, family, and school characteristics. Of 10,724 participants, approximately 50% identified as female, 63% White, 21% Black, 17% Latinx. School quality indicators were not statistically associated with adult depressive symptoms for White participants. Teacher retention was unrelated to self-rated health for any group, but was associated with greater depression symptoms in adulthood for Black and Latinx participants (β = 0.42-point higher score per 1 SD increase in teacher retention, 95% CI [0.153, 0.690], *p* = 0.002; β = 0.23-point higher score per 1 SD increase in teacher retention, 95% CI [0.003, 0.466], *p* = 0.047, respectively). Some conventional school quality indicators were associated with worse adult mental health for Black and Latinx participants. The findings suggest that more research is needed to understand what aspects of school quality may best promote life course health development for minoritized groups.

## Introduction

Although an established literature links years of schooling to improved academic and health outcomes [1–5], a growing evidence underscores the importance of school quality and the multiple domains through which it may be assessed [4, 6]. For example, four distinct components of school quality (student promotion rates, teacher retention, student attendance, and parental involvement) have been shown to longitudinally predict both academic achievement and young adult health in a general population [5]. Yet little research has examined whether these associations differ by race and ethnicity.

School quality may improve life course health through multiple avenues including: (a) cognitive skills (e.g., enhancing health literacy), (b) non-cognitive skills and behaviors (e.g., substance use), (c) social connections (e.g., mentoring, job finding, social support), and (d) linkage with healthcare (e.g., individualized education plans) [5, 7–9]. Higher quality schooling may also promote engagement and persistence in education which further enhances downstream benefits [4], enabling individuals to manage health more effectively [10]. However, these benefits may depend on access to complementary resources (e.g., social status). One way to conceptualize this relationship is through resource multiplication theory, which posits that education amplifies existing advantage, such that health returns are greater for socially advantaged groups and attenuated for those whose access to complementary resources is constrained [11]. Despite clear benefits of schooling to a general population, key gaps remain: (a) the degree to which health benefits may differ depending on social context, particularly race and racism and (b) the optimal indicators for measuring school quality with the goal of promoting health and equity [4].

While high quality schooling is presumed to promote health, contemporary evidence suggests that upward educational and income mobility may improve health for White Americans but produce no health benefits or even come at a cost for Black and other minoritized Americans [12–15]. These patterns may reflect structural racism operating cumulatively across the life course, whereby unequal educational experiences and broader social conditions shape divergent health trajectories. From this perspective, the extent to which school quality translates into improved adult health may vary systematically across racial and ethnic groups as socially modified factors accumulate throughout the life span and set individuals on trajectories of improving or deteriorating health development.

Given the known importance of school quality for academic outcomes and its association with healthy development across the life course [5], we sought to evaluate longitudinal associations between indicators of school quality during middle and high school and downstream adult health for different racial and ethnic groups. Given the aforementioned disparities in health despite socioeconomic and educational attainment, we hypothesized that school quality would be associated with better self-reported health in adulthood to a greater degree among adult who identify as White than it is among those who identify as Black or Latinx.

## Method

### Participants

We analyzed data from the National Longitudinal Study of Adolescent Health (Add Health), a nationally representative cohort study. In 1994–1995, Add Health recruited 20,745 adolescents in grades 7–12 using a stratified random sample from 80 high schools and 65 feeder schools [16]. Five waves of data have been collected including baseline Wave I (1994–1995, ages 12–19), Wave III (2001–2002, ages 19–26), Wave IV (2007–2008, age 24–32), and the most recent Wave V (2016–2018, ages 32–42). Wave I included data collection in schools, in homes, and administrator surveys administered to 79 of 80 high school administrators who reported school-level characteristics. At Wave V, 19,828 individuals were living and eligible for follow-up [16, 17].

We restricted our sample to participants who (a) had at least one school quality indicator, (b) had complete data on the primary outcome, (c) attended one of the 132 schools included in the Add Health Wave I sample with administrator surveys, (d) had valid sample weights, and (e) self-identified as White, Black, or Latinx. Our analytic sample thus included 10,724 participants from 132 schools. A flow diagram of the sample selection process can be found in the supplement (Figure A1).

### Indicators of School Quality

Historically, school quality has been variably defined using a myriad of measures [4, 18, 19]. Based on literature review, we selected the following four school quality indicators that, based on previous longitudinal analyses of Add Health, are associated with positive academic outcomes (i.e., high school graduation, college attendance) and health outcomes (i.e. self-rated health, depression, obesity) [5]. Each of these indicators was reported at the school-level on the school administrator’s survey.

#### Student Promotion Rates

For each school, we calculated the percentage of students who were promoted to the next grade or who completed high school. We then standardized these school-level student promotion rates across schools using z-scores, with one unit change equal to one standard deviation.

#### Teacher Retention

School administrators reported the percentage of teachers who were full-time and had been employed at the school for five years or more. This measure gives an idea both of teacher experience in the classroom and a school’s teacher turnover, where a greater percentage of experienced teachers is advantageous [20]. For ease of interpretability, we standardized this value using z-scores.

#### Average Daily Attendance

Based upon the response distribution and consistent with prior analyses [5], we consolidated the original 5-category variable as reported by school administrators into three levels of average daily attendance: 75–89% (low), 90–94% (medium), and ≥ 95% (high).

#### Parental Involvement

Add Health captures parental participation in school organizations, such as Parent-Teacher Associations, as reported by a school administrator. There is no generally accepted procedure in the literature for categorization of this variable. Prior literature has examined school-level parental involvement in Add Health data using different cut-points and as a continuous variable [5]. Therefore, we replicated these same categories: No parental organization, 0–14% parental participation (low), 15–29% participation (medium), and 30–100% participation (high).

### Academic Outcomes

To provide evidence of validity for the use of these measures in the present analyses, we confirmed that each school quality indicator was positively associated with high school graduation, college attendance, or both in race-stratified analyses (Appendix Table A2). At Wave III, Add Health collected student high school transcripts which designated whether students received a high school diploma, GED, or no diploma. At Wave IV participants reported their highest level of education including four levels of college attendance (i.e., no college, some college, college completion, and post-collegiate education). In previous, unstratified analyses, each of the four school quality measures was consistently associated with these academic outcomes [5]. For this race-stratified validation analysis, we created two primary academic outcome variables: (a) receiving a high school diploma or GED versus neither and (b) attending any college versus none.

### Health Outcomes

We chose our primary outcome of general health for its utility in predicting present and future health [21] as well as its association with morbidity and functional ability, health care utilization, and mortality [22–29]. General health is also an important outcome for race-based health disparities research [30]. We chose depression as it is one of the most prevalent mental health problems in the USA [31] and is characterized by racial disparities including increased rates of depression for those suffering racial discrimination, lower overall prevalence but greater chronicity of symptoms for people of color, and poorer access to care for people of color [12, 32, 33]. In ancillary analyses (see Appendix for methods and results), we chose additional mental and cardiometabolic secondary outcomes given the disproportionate burden of these conditions among Black and Latinx groups compared to their White counterparts and the literature on diminishing returns [12].

#### Self-Rated Health

In the Add Health study, participants reported their general health as excellent, very good, fair, or poor in Waves I and V, which was modeled as a continuous variable.

#### Depressive Symptoms

In Waves I-V, participants responded to the Center for Epidemiologic Studies Depression Scale (CES-D) short form. The CES-D is a widely used 10-item screening tool for depressive symptoms in the previous 7 days and has strong predictive validity for frequency and severity of contemporaneous depression [34, 35]. Each item has four levels of frequency from 0 (*rarely*, *0 days*) to 3 (*severe*, *5–7 days*). Wave V asked participants to respond to the 5-item negative affective subscale of the CES-D which has been independently validated for minoritized populations [36]. Using this subscale, we summed the frequencies of each item creating a severity scale from 0 to 15 as has been done previously [36, 37].

### Strata and Covariates

#### Race

We constructed race from Add Health self-reported data. In Wave I, Add Health asked participants “Are you of Hispanic origin?” (yes/no) and “What is your race?” (Mark one or more boxes: “White,” “Black/African,” “Hispanic/Latino,” “Asian,” “Pacific Islander,” “American Indian/Alaska Native,” and “Other”). Participants were classified as Hispanic if they affirmatively responded to the former question, regardless of their selection in the latter question. We limited our analysis to (non-Hispanic) White, (non-Hispanic) Black/African, and Hispanic/Latino individuals due to sample size limitations and because of well-documented disparities between these groups. Participants who elected more than one racial identity were excluded given our limited ability to characterize forms of intersectionality with this study design. Hereafter the categories of Black/African and Hispanic/Latino will be referred to as Black and Latinx, respectively.

#### Covariates

*A priori* covariates included factors that could potentially confound the association between school quality on adult health at the individual level (i.e., baseline self-reported health, age, sex, household income, parental education, family structure, and household language as a measure of acculturation) and school level (i.e., school size, school type, school urbanicity, school region, middle school vs. high school), all collected during Wave I. Table 1 describes the categorization of each variable.

### Data Analysis

Our analyses followed the same general approach as prior longitudinal analyses of the association between school quality and health [5]. We used survey weights to account for the three survey design elements: stratification, clustering, and weighting. We utilized a single-stage design with clustering at the school level. All analyses were stratified by the three racial and ethnic groups detailed above.

#### Association of School Quality Indicators with Academic Outcomes

We modeled each academic outcome as a function of each school quality indicator using logistic regression stratified by race/ethnicity and adjusting for individual- and school-level covariates.

#### Main Analyses

In our primary and ancillary analyses, we used linear regression models for continuous outcomes and logistic regression models for dichotomous outcomes to test whether each Wave I school quality indicator was associated with Wave V health outcome adjusting for the same covariates listed above and with the addition of Wave I (baseline) health outcome. All models used Wave V survey weights. To quantify effect modification by race/ethnicity, we also modeled each outcome as a function of each school quality indicator with an interaction term for the school quality and race/ethnicity, adjusting for confounders as above. Analyses of additional health outcomes, sensitivity analyses using ordinal logistic regression, and post-hoc analyses examining potential mediators are presented in the Appendix Methods. All analyses were performed using STATA version 14.2 (Stata Corp, College Station, TX).

## Results

### School and Participant Characteristics

Demographics of our analytic sample were similar to the overall Add Health sample, however, statistically significant differences between included and excluded participants were present (Appendix Table A1). Included participants were more likely to be female, White, have a college degree, higher household income, two biological parents, and speak English as the household language. They also reported slightly better self-reported health and fewer depressive symptoms at baseline.

Table 1 shows the analytic sample stratified by race/ethnicity. Individuals from all three race and ethnicity categories were similar in their mean age at baseline and identifying as female or male. White participants were most likely to have a parent with greater than a high school diploma, whereas Latinx participants were most likely to have parents with less than a high school diploma. White participants were also most likely to have greater household income and two biological parents in the household compared to Black and Latinx participants. Approximately 57.4% of Latinx participants reported speaking English as their primary household language as compared to White (99.4%) and Black (99.8%) participants.

Most Latinx participants (60.0%) attended large schools of greater than 1,000 participants, whereas a plurality of White (46.6%) and Black (49.8%) participants attended medium sized schools of 401–1000 students. Most participants (93.5%) attended public schools. Most White and Black participants attended suburban schools (63.6 and 52.2%, respectively). Approximately one-third of Black and nearly two-thirds of Latinx participants attended urban schools. Approximately a third of white participants attended schools in the Midwest (38.5%), most Black participants attended a school in the South (65.7%) and Latinx participants in the West (34.2%).

Table 1 reports school quality indicators stratified by race. All quality indicators, apart from teacher retention, varied significantly between race/ethnicity categories. Mean student promotion was lower among Black and Latinx participants’ schools compared to White participants (89.4%, 90.5%, 94.7%, respectively). Average daily attendance was estimated to be above 95% at schools attended by 42.2% of White participants, 29.2% of Black participants, and 26.6% of Latinx participants. Approximately 14.2% of schools reported no parent-teacher organization and this was most common among schools attended by White participants. Parent involvement was estimated to be greater than 30% at schools attended by 23.9% of White participants, 30.3% of Black participants, and 23.8% of Latinx participants. Correlations between school quality indicators were less than 0.5. Although White participants were more likely to attend schools with higher quality indicators, our analysis of the distribution of school quality by race confirmed sufficient overlap to support comparative evaluation.

Health outcomes in wave V also varied by race strata (Table 2), except for depressive symptoms and suicidality. Compared to Black and Latinx participants, White participants tended to have better self-reported health, greater prevalence of depressive diagnosis, and lower prevalence of hypertension, diabetes, and cardiovascular diagnoses.

### School Quality and Academic Outcomes

Each school quality indicator was associated with either high school graduation or college attendance in stratified analyses (Appendix Table A1). The exception to this pattern was teacher retention, which was only marginally significantly associated with high school graduation for White participants in adjusted models.

### School Quality and Adult Health

#### Self-rated health (Fig. 1.A., Appendix Table A3).

School quality indicators were not statistically associated with self-rated health for White and Latinx participants. Among Black participants, higher student retention in high school was associated with worse adult self-rated health (i.e., higher health score) but the association was only marginally significant (*β* = 0.08 increase in self-rated health for each standard deviation increase in teacher retention, 95% CI [0.000, 0.152], *p* = 0.050). In accompanying models examining interaction terms between race/ethnicity and school quality indicators, no interaction terms were statistically significant.

#### Depressive symptoms (Fig. 1.B., Appendix Table A3).

School quality indicators were not statistically associated with adult depressive symptoms for White participants. Yet, higher teacher retention was associated with a greater CES-D depressive symptom score in adulthood among Black participants (*β* = 0.42 greater depressive symptom score reported for each standard deviation increase in teacher retention, 95% CI [0.153, 0.690], *p* = 0.002). Similarly, moderate (vs. high) parental involvement (was associated with lower CES-D depressive symptom score among Black participants (*β* = −0.59, 95% CI [−1.181, −0.004], *p* = 0.049). For Latinx participants, higher teacher retention was also associated with higher depressive symptom score (*β* = 0.23, 95% CI [0.003, 0.466], *p* = 0.047). Finally, low (vs. high) average daily attendance was associated with greater adult depressive symptom score for Latinx participants (*β* = 0.98, 95% CI [0.258, 1.697], *p* = 0.008). In models using an interaction term between the school quality indicator and race/ethnicity, the interaction terms between teacher retention and White-Black as well as White-Latinx race/ethnicity were significant (*p* = 0.017, *p* = 0.033, respectively). The interaction term between parental involvement at the 15–29% level and White-Black race/ethnicity was significant (*p* = 0.004). The adjusted Wald test of interaction effect was not significant for attendance-race/ethnicity (*p* = 0.107).

#### Ancillary analyses (Appendix Table A3).

Ancillary analyses modeling additional mental health and cardiometabolic outcomes find similar patterns for mental health outcomes including suicidal ideation, in which some school quality indicators are only associated with improved health for White participants or may even be associated with poorer health outcomes for Black and Latinx participants in adulthood.

## Discussion

To our knowledge, these data are the first to examine how indicators of school quality relate to health in adulthood among racially and ethnically diverse groups. Overall, our analysis showed mixed results. School quality indicators were not strongly associated with self-reported health for any race/ethnicity group. Higher teacher retention was associated with worse adult depressive symptoms among Black and Latinx participants. Low daily attendance was associated with greater adult depressive symptoms among Latinx participants but not for Black or White participants. Although moderate (vs. high) parental involvement was associated with lower depressive symptoms among Black participants, this pattern was not observed for the other two race/ethnicity groups. In contrast, prior unstratified analyses have demonstrated positive or null associations between these same school quality indicators and long-term health outcomes [5]. The discrepancy suggests that previous findings may have been driven by the larger White subgroup. Our findings suggest that different dimensions of school quality likely contribute to the health benefits of education in divergent ways. Accordingly, these dimensions warrant separate consideration before joint consideration, particularly across different racial and ethnic groups.

While sparse literature examines school quality indicators and health by race/ethnicity, existing studies provide corroborating evidence that academic achievement and health may become decoupled for minoritized groups [13, 38–43]. Evidence from the desegregation of schools and from historically Black colleges and universities support the contentions that addressing conventional school quality measures alone will not close racial/ethnic health gaps but that some specific social contexts may be particularly well suited to reduce disparities [7, 44]. This study’s findings diverge with this literature primarily on self-reported health and physical health outcomes. Instead of finding that better school quality was associated with poorer self-reported health and physical health outcomes for racial/ethnic groups [41, 45–47], we found no association with self-reported health and marginal if any associations with cardiometabolic health outcomes (e.g., cardiovascular disease, diabetes, hypertension, hyperlipidemia) (see Appendix). The absence of such findings may be due to several factors, including that: (a) we examined school quality indicators which are related to academic achievement but not the more specific characteristic of upward mobility as many of these prior studies operationalize, (b) the timing of outcomes measured (i.e., Wave V) occurred at a younger age than the peak incidence for many cardiometabolic health outcomes [48–50], and (c) we were unable to account for specific characteristics of previous cohorts such as geographic locations, occupations, biopsychosocial inheritances, and community histories, all of which influence risk of psychosocial stress and health behaviors.

Although the school quality indicators of this study showed longitudinal associations with positive academic outcomes, associations with health outcomes were mixed for different racial/ethnic strata. We are unable to draw specific inferences as to why some school quality measures appear to have a stronger association with health using this dataset; however, the findings raise questions about whether some quality measures may be more important or play different roles for specific communities. Future studies could investigate a more diverse array of candidate indicators. Empirical evidence suggests that connectedness to school [51], connectedness to teachers, presence of role models [52], student-teacher racial/ethnic concordance [53], extracurricular offerings [54], integrated support services [55], and other indicators may be of particular importance for Black and Latinx youth and their communities in the USA. Future comparative observational studies and community-based participatory interventions may help school leaders and public health practitioners identify the aspects of school quality that may be most beneficial to specific communities.

Detailed examination of the mechanisms by which race/ethnicity may alter education’s impact on health are not possible with these data. Yet these findings and the adjacent literatures on diminishing returns [12] and skin-deep-resilience [13] support a resource-multiplication theory of educational attainment in which the full health benefits of education may depend on complementary resources that systemic racism constrains [11]. When the utility of one resource (e.g., education) depends on another (e.g., social status), individuals lacking those complements may derive less benefit. In future research, this may become a useful framework for understanding the broader relationship between education and mental health. Using a biopsychosocial model of health, we hypothesize potential mechanistic categories informed by current understanding of how racism impacts individuals across the life course, including: (a) pathophysiologic changes from psychosocial stressors (e.g., minoritized groups may face both greater likelihood of discrimination when upwardly mobile into White-dominated settings for work and higher education, ultimately leading to cortisol dysregulation, inflammation, allostatic load, and possibly shortened telomeres) [41, 56–59], (b) development of health-related practices or beliefs (e.g., Black Americans may be more likely to use substances like tobacco as a method of coping with discrimination-associated chronic stress) [60–63], (c) reduction of protective factors (e.g., minoritized groups report greater social isolation when upwardly mobile) [58, 64], and (d) many mobility benefits may be absent or reversed for specific minoritized groups (e.g., upwardly mobile minoritized groups may be less likely to move to more affluent neighborhoods and some desirous residential areas, such as suburbs, may actually be associated with better health for White Americans and worse health for Black Americans) [65–67]. When considering the role of school quality specifically, these mechanisms may be classified by stage of life course: (a) factors that develop while in attendance at a high-quality school (e.g., meaning-making environments that lead to maladaptation with later life consequences) [68–70], (b) factors that occur after graduation in the transition to higher education or the job market (e.g., consolidation of substance use or coping maladaptation) [60, 71], (c) factors present in the workforce (e.g., differentially increased experience of discrimination) [38, 72], and (d) factors that are present throughout the life course but may be enhanced by the social environments of upwardly mobile minoritized individuals (e.g., chronic stress resulting in increased allostatic load) [56, 59, 73, 74].

We found a similar but distinct pattern of associations for individuals identifying as Latinx compared to Black. This may be due to differential patterns of racism nationally [73, 74], awareness and perception of racism based on an individual’s degree of acculturation and English proficiency [74–76], or simply due to the underlying heterogeneity of the Latinx community who represent many different subethnic groups, phenotypes, nationalities, cultures, ancestries, and social histories. Our exploratory mediation analyses did not reveal any patterns of mediation by college attendance or perceived disrespect, however, given the multitude of ways that racism may act on health, this is not unexpected and should be investigated with more diverse and precise measurements of racism.

### Strengths and Limitations

There are several notable strengths of this work. First, few studies of its kind leverage nationally representative data of adolescents growing into adulthood, a critical element of life course research. Second, various measures of school quality have been proposed and while there is no set of standardized, comprehensive measures, we chose four diverse measures previously shown to predict two important longitudinal academic outcomes: high school graduation and college attendance [5]. Furthermore, we then reassessed their associations with these academic outcomes in race-stratified analyses. Third, many studies of health disparities only compare Black and White Americans. In this study, we were able to also include at least one ethnic category to further generalize our findings.

Several limitations are also worth noting. First, we cannot exclude the possibility of residual confounding despite accounting for multiple potential confounders at the individual, family, and school levels. To mitigate this risk, we verified substantial overlap in schools attended across racial/ethnic groups, reducing confounding by structural segregation, and the longitudinal design helps limit reverse causality. Nevertheless, population-level analyses remain susceptible to unmeasured confounding, and we cannot fully adjust for all factors (e.g., differential school funding that allocates greater resources to higher-need schools). We are also unable to deconstruct the many elements that make up the conventional school quality indicators presented (e.g., teacher retention as either representing greater teacher experience or school retention efforts) or the mediation pathways through which quality indicators may be associated with health (e.g., differential stressors). Second, our analyses are limited to a single generation of Americans and cannot properly test for secular trends. Third, there may be measurement bias when implementing the same survey across racial and ethnic groups. For example, some evidence suggests that Latinx adults may systematically report lower self-rated health compared to non-Latinx adults – although the association between self-rated health and morbidity and mortality remains strong [77]. Although we adjusted for baseline health in all our models, we are unable to account for biases in self-report that may change over the life course. Fourth, race/ethnicity may be defined and operationalized differently depending on sociocultural and historical context. Our definition of race/ethnicity and stratification into three race/ethnicity categories was a limitation imposed by statistical constraints as well as by the Add Health dataset which adhered to prevailing standards when the study was conceived [78, 79]. Of note, we were unable to include individuals who identified as multiracial or those for whom race/ethnicity was not available. The racial and ethnic groups do not capture the many facets of racism and thus our ability to generalize and test specific mechanisms by which disparities appear are limited. Finally, the associations identified are correlational and causality cannot be inferred.

## Conclusion

In conclusion, some measures of school quality were associated with worse adult mental health for racially and ethnically minoritized groups in this nationally representative, longitudinal cohort of adolescents growing into adulthood, suggesting that the lifelong health benefits associated with conventional indicators of school quality are not shared equally by all students. Additional research is needed to better understand what aspects of the school experience may support both the short-term goal of facilitating educational attainment and the long-term goal of achieving health equity over the life course. Such factors might inform the development of novel school quality indicators and interventions to mitigate the negative effects of racism and discrimination on health. Recognizing that improving school quality for all students remains an important goal, educators, public health practitioners, and policymakers are faced with the question of how best to cultivate educational attainment and human capital for continued health development in and beyond adolescence.

## Supplementary Material

Supplemental Material

**Supplementary Information** The online version contains supplementary material available at https://doi.org/10.1007/s40615-026-03118-4.

## Figures and Tables

**Fig. 1:**
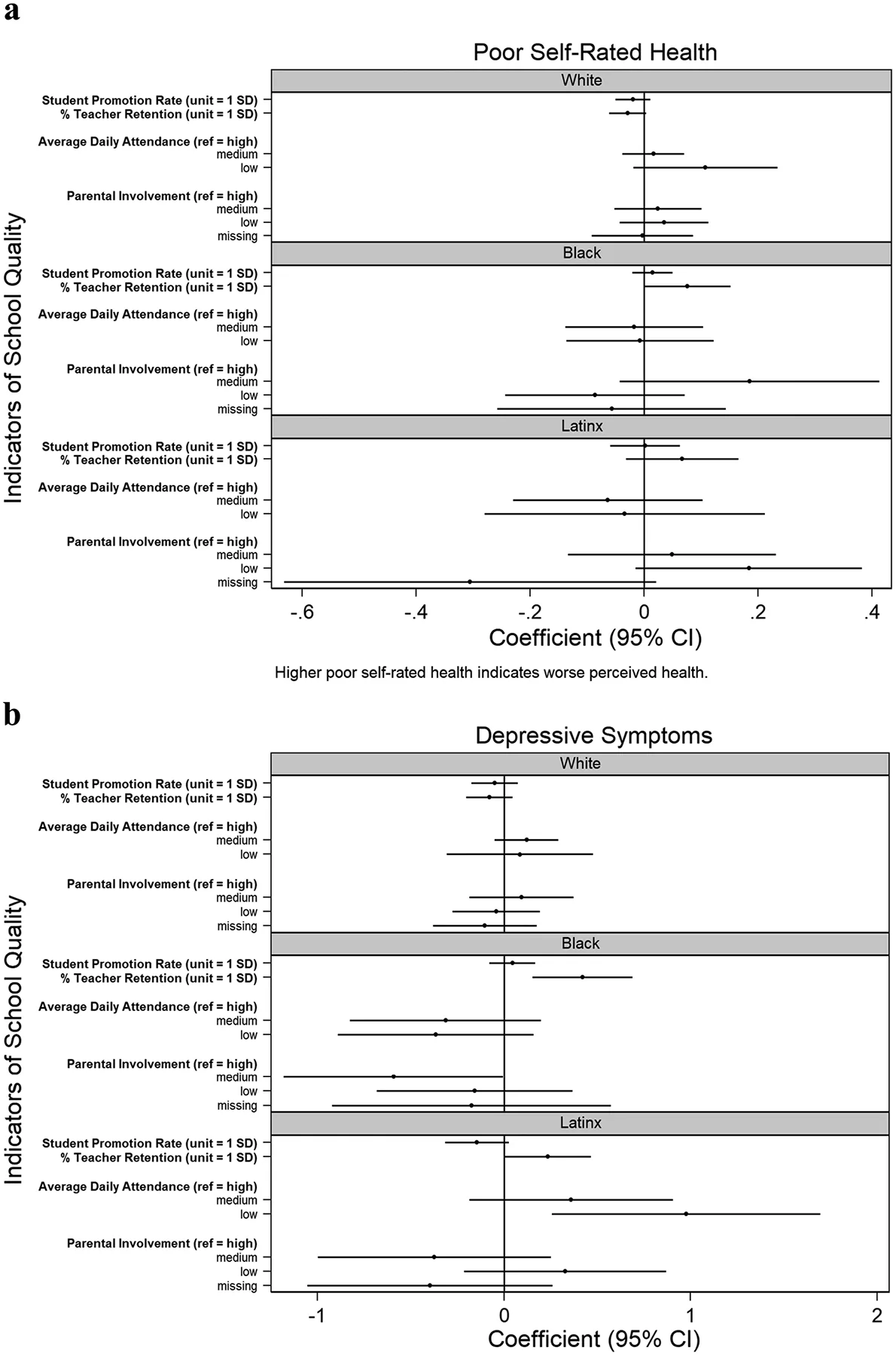
Associations between school quality measures (Wave I) and health outcomes (Wave V), stratified by participant race/ethnicity, National Longitudinal Survey of Adolescent Health 1994–2008. Upper panel (**a**): Lower health score indicates better self-rated health. Lower panel (**b**): Higher CES-D score indicates greater symptoms of depression. Models are adjusted for age, sex, household income, parent education, family structure, household language, school size, school type, school urbanicity, school region, school level (middle/high), and health outcome at baseline. Note on interactions: In the upper panel, no school quality indicator-race/ethnicity interaction was statistically significant. In the lower panel, the interaction term between White and Black and White and Latinx were significant for teacher retention (*p* = 0.017 and *p* = 0.033, respectively), the interaction term between White and Black for 15–29% parental involvement was significant (*p* = 0.004)

**Table 1:** Sample demographics (Wave I), school quality measures (Wave I), and health outcomes (Wave V), National Longitudinal Survey of Adolescent Health, 1994–2008

	OverallNo. (Weighted Percentage)(*N* = 10,724)	WhiteNo. (Weighted Percentage)(*n* = 6,718)	BlackNo. (Weighted Percentage)(*n*=2,225)	LatinxNo. (Weighted Percentage)(*n* = 1,781)	*p*-value^a^
Demographics (Wave I)					
Individual Demographics					
Age, mean (SD)	15.4 (1.8)	15.4 (1.7)	15.6 (2.0)	15.5 (2.1)	0.585
Gender					0.859
Female	6,091 (49.7)	3,705 (49.6)	1,375 (50.5)	1,011 (49.5)	
Male	4,633 (50.2)	3,013 (50.4)	850 (49.5)	770 (50.5)	
Household Demographics					
Highest parent education					< 0.001
Less than high school	996 (9.0)	323 (5.6)	156 (8.1)	517 (29.4)	
High school diploma	3,065 (31.6)	1,909 (30.2)	669 (39.0)	487 (30.5)	
Some college	2,312 (22.1)	1,531 (23.0)	455 (21.4)	326 (18.1)	
College degree or more	4,095 (37.2)	2,864 (41.2)	862 (31.5)	369 (22.0)	
Household income					< 0.001
< 25 K	2,268 (22.2)	1,030 (17.0)	690 (35.5)	548 (33.7)	
25–49 K	2,761 (25.9)	1,848 (27.3)	483 (22.1)	430 (23.1)	
50–74 K	1,997 (19.2)	1,557 (22.9)	253 (9.4)	187 (11.0)	
≥ 75 K	1,271 (11.2)	1,021 (13.8)	150 (4.3)	100 (5.5)	
Missing	2,427 (21.5)	1,262 (18.9)	649 (28.7)	516 (26.6)	
Family structure					< 0.001
Two biological parents	5,757 (55.9)	4,025 (61.4)	749 (31.5)	983 (55.7)	
Two parents (≥ 1 non-bio parent)	1,879 (16.2)	1,309 (17.3)	306 (12.5)	264 (15.0)	
Single parent	2,527 (22.5)	1,151 (17.5)	955 (44.0)	421 (23.4)	
Other	561 (5.4)	233 (3.8)	215 (11.9)	113 (5.9)	
Household language					< 0.001
English	9,873 (93.9)	6,679 (99.4)	2,217 (99.8)	977 (57.4)	
Spanish	787 (5.4)	2 (0.0)	3 (0.1)	782 (41.0)	
Other	64 (0.6)	37 (0.5)	5 (0.1)	22 (1.7)	
School Characteristics					
School size					0.054
Small (1–400)	1,623 (16.8)	1,193 (18.2)	325 (16.2)	105 (10.2)	
Medium (401–1000)	4,173 (44.9)	2,875 (46.6)	951 (49.8)	347 (29.9)	
Large (1001–4000)	4,928 (38.3)	2,650 (35.2)	949 (34.1)	1,329 (60.0)	
School type					0.145
Public	9,921 (93.5)	6,096 (92.5)	2,106 (96.2)	1,719 (95.6)	
Private	803 (6.5)	622 (7.5)	119 (3.8)	62 (4.4)	
Urbanicity					< 0.001
Urban	3,203 (26.4)	1,474 (18.2)	734 (33.3)	995 (61.6)	
Suburban	5,612 (58.0)	3,685 (63.6)	1,201 (52.2)	726 (35.1)	
Rural	1,909 (15.7)	1,559 (18.3)	290 (14.5)	60 (3.3)	
Region					< 0.001
West	2,040 (14.2)	1,015 (12.4)	287 (5.6)	738 (34.2)	
Midwest	2,911 (32.1)	2,377 (38.5)	405 (21.4)	129 (10.9)	
South	4,231 (40.3)	2,191 (34.6)	1,376 (65.7)	664 (39.8)	
Northeast	1,542 (13.5)	1,135 (14.6)	157 (7.3)	250 (15.1)	
School Quality Measures (Wave I)					
Student promotion, mean (SD)	93.2 (7.1)	94.7 (5.3)	89.4 (9.9)	90.5 (10.4)	< 0.001
Teacher retention, mean (SD)	67.3 (20.6)	68.3 (20.2)	64.4 (18.6)	65.9 (22.6)	0.449
Average daily attendance					< 0.001
High	3,558 (38.0)	2,607 (42.2)	608 (29.2)	343 (26.6)	
Medium	5,047 (47.9)	3,427 (50.1)	809 (36.2)	811 (50.5)	
Low	1,958 (14.1)	542 (7.7)	797 (34.6)	619 (22.9)	
Parent involvement					0.017
High	2,693 (24.9)	1,666 (23.9)	729 (30.3)	298 (23.8)	
Medium	2,040 (21.7)	1,465 (23.5)	238 (11.0)	337 (25.5)	
Low	4,756 (39.1)	2,563 (35.1)	1,130 (52.1)	1,063 (45.0)	
No PTA	1,235 (14.2)	1,024 (17.6)	128 (6.6)	83 (5.8)	
Baseline Health (Wave I)					
Self-reported health					< 0.001
Excellent	3,113 (29.2)	1,900 (28.3)	728 (33.4)	485 (28.9)	
Very good	4,315 (39.8)	2,828 (41.6)	828 (36.4)	659 (34.3)	
Good	2,618 (25.0)	1,613 (24.4)	517 (23.3)	488 (30.6)	
Fair	629 (5.5)	351 (5.2)	142 (6.7)	136 (5.5)	
Poor	48 (0.5)	25 (0.5)	10 (0.3)	13 (0.8)	
Depressive symptoms, mean (SD)	6.4 (4.6)	6.1 (4.2)	7.0 (5.2)	7.2 (5.3)	< 0.001
BMI, mean (SD)	22.6 (4.4)	22.2 (4.0)	23.5 (5.5)	23.1 (5.0)	< 0.001
Suicidal ideation					0.038
Yes	1,430 (13.4)	935 (14.1)	250 (10.7)	245 (13.2)	
No	9,210 (86.6)	5,741 (85.9)	1,954 (89.3)	1,515 (86.8)	

*Abbreviations:SD* Standard Deviation, *BMI* Body Mass Index

a We used an adjusted Wald F test to determine whether there were differences in means across race/ethnicity for continuous variables and a design-based F test to determine whether there were associations between race/ethnicity and categorical variables

**Table 2:** Sample health outcomes (Wave V), National Longitudinal Survey of Adolescent Health, 1994–2008

	OverallNo. (Weighted Percentage)(*N* = 10,724)	WhiteNo. (Weighted Percentage)(*n* = 6,718)	BlackNo. (Weighted Percentage)(*n* = 2,225)	LatinxNo. (Weighted Percentage)(*n* = 1,781)	*p*-value^a^
Self-reported health					< 0.001
Excellent	1,796 (16.3)	1,196 (17.3)	307 (12.8)	293 (15.4)	
Very good	3,899 (36.4)	2,604 (38.3)	689 (29.6)	606 (34.5)	
Good	3,592 (33.4)	2,147 (32.3)	841 (38.1)	604 (33.2)	
Fair	1,192 (11.5)	631 (9.6)	338 (17.3)	223 (14.4)	
Poor	245 (2.5)	140 (2.5)	50 (2.3)	55 (2.5)	
Depressive symptoms, mean (SD)	2.4 (2.6)	2.4 (2.4)	2.6 (3.2)	2.4 (2.9)	0.119
Depression diagnosis					< 0.001
Yes	2,695 (26.4)	1,928 (29.0)	414 (19.0)	353 (21.2)	
No	7,985 (73.6)	4,766 (71.0)	1,798 (81.0)	1,421 (78.8)	
High cholesterol diagnosis					0.084
Yes	1,827 (16.7)	1,129 (16.2)	370 (16.7)	328 (19.4)	
No	8,834 (83.3)	5,555 (83.8)	1,837 (83.3)	1,442 (80.6)	
Hypertension diagnosis					< 0.001
Yes	2,023 (19.3)	1,130 (17.5)	596 (27.8)	297 (18.2)	
No	8,646 (80.7)	5,556 (82.5)	1,615 (72.2)	1,475 (81.8)	
Diabetes diagnosis					< 0.001
Yes	641 (5.7)	299 (4.4)	210 (10.7)	132 (6.1)	
No	10,038 (94.3)	6,394 (95.6)	2,003 (89.4)	1,641 (93.9)	
Cardiovascular diagnosis					< 0.001
Yes	193 (1.9)	106 (1.5)	57 (4.0)	30 (1.6)	
No	10,416 (98.1)	6,556 (98.5)	2,129 (96.0)	1,731 (98.4)	
Suicidal ideation					0.670
Yes	672 (6.6)	437 (6.8)	139 (6.4)	96 (5.9)	
No	9,814 (93.4)	6,159 (93.2)	2,015 (93.6)	1,640 (94.1)	

a We used an adjusted Wald F test to determine whether there were differences in means across race/ethnicity for continuous variables and a design-based F test to determine whether there were associations between race/ethnicity and categorical variables
