# Supplementary material for: Racial Differences in the Association Between School Quality and Adult Health: A Nationally Representative Longitudinal Study
Source: J Racial Ethn Health Disparities. Author manuscript; Available in PMC 2026 Nov 14. (PMC13769428; doi:10.1007/s40615-026-03118-4)
Supplement: Supplementary file 1 [file NIHMS2219697-supplement-Supplemental_Material.docx]

# Appendix

## Methods

### Additional Health Outcomes

*Self-reported depression diagnosis* – In wave I-V, participants reported whether they had ever been diagnosed with depression by a health care professional. Depression diagnosis was analyzed as a dichotomous yes/no variable.

*Suicidal Ideation* – Suicide is a devastating consequence of mental illness and represents a closed door for intervening to help that individual. Participants in Wave V responded to if they had “seriously thought of about committing suicide” in the preceding 12 months. We analyzed this as a dichotomous variable.

*Hypertension* – Chronically elevated arterial blood pressure is associated with a broad range of cardiovascular and renal morbidity and mortality. In Wave V, participants were asked if a “doctor, nurse, or other healthcare provider every told you that you have or had high blood pressure or hypertension [female: when you were not pregnant.]”. We analyzed this as a dichotomous variable.

*Diabetes* – Similarly, diabetes – defined as a disorder of glucose metabolism that results in elevated levels of blood glucose – results in a host of negative health outcomes if left untreated including vision impairment and blindness, heart attacks and strokes, nerve damage, and cancer, ultimately impairing function and leading to morbidity and mortality. In Wave V, participants were asked if a “doctor, nurse, or other health care provider ever told you that you have or had high blood sugar or diabetes [female: When you were not pregnant.]” We analyzed this as a dichotomous variable.

*Hyperlipidemia* – Hyperlipidemia and elevated non-high density lipoprotein (non-HDL) is a major predictor of atherosclerotic cardiovascular disease, a leading cause of morbidity and mortality worldwide. In Wave V, participants responded to “Has a doctor, nurse, or other health care provider ever told you that you have or had high blood cholesterol or triglycerides or lipids?” which was analyzed as a dichotomous variable.

*Cardiovascular Disease* – Cardiovascular disease, which include coronary heart disease, cerebrovascular disease and others, is the leading cause of death worldwide.^79^ Participants in Wave V of Add Health reported if a doctor, nurse or other healthcare provider had ever diagnosed them with heart disease, heart failure, or stroke respectively in three separate questions. These three conditions collectively constitute the majority of cardiovascular disease.^80^ We created a dichotomous composite variable of any or none of these three conditions.

### Post-hoc Mediation Analyses

*Post-hoc analyses* – Our post-hoc mediation analyses were designed to test two possible mechanisms for the associations we found in our main analyses: differential exposure to racial discrimination and exposure to racism in higher education. These analyses were premised on (a) the educational attainment observed from attending a high quality school that increases the likelihood of educational or socioeconomic upward mobility and (b) prior research indicating that minoritized individuals who achieve such upward mobility enter cultural spaces where they experience more frequent and severe forms of some types of racial discrimination as well as be more aware of discrimination than their counterparts who lack this mobility.^11,70,81^ In Wave IV, Add Health collected data on interpersonal racial discrimination in two questions: 1) “In your day-to-day life, how often do you feel you have been treated with less respect or courtesy than other people?” answer choices: “never,” “rarely,” “sometimes,” “often,” “refused,” “I don’t know” and 2) “What do you think was the main reason for being treated with less respect or courtesy than other people?” These two questions were combined into a three-category ordinal variable representing the report of experiencing less respect/courtesy because of “race,” “ancestry or national origin,” or “shade of skin color” either: never, rarely, sometimes (0), a lot of the time (1), or most of the time/all of the time (2) (answer choices were also dichotomized to never, rarely, sometimes vs. a lot of the time, most of the time, and all of the time in sensitivity analyses that yielded the same results). In our second post-hoc analysis, we used participant matriculation in a four-year college as self-reported in Wave III as a mediator in our models. We modeled outcomes introducing each mediator as a covariate using standard methods.^82^

*Sensitivity Analyses*

To examine the robustness of the findings, we estimated ordinal logistic regression models for the two primary outcomes (Appendix Table A4). All other model specifications including covariates, stratification, clustering, and weighting were identical to those used in the primary analyses.

## Results

### Tables

| **Table A1. Comparison of baseline (Wave 1) characteristics between included and excluded participants.** | | | | |
| --- | --- | --- | --- | --- |
|  | **Overall**  No. (Weighted Percentage)  (*N*=20,745) | **Excluded**  No. (Weighted Percentage)  (*N*=10,021) | **Included**  No. (Weighted Percentage)  (*N*=10,724) | ***p*-value^a^** |
|  |  |  |  |  |
| **Age, mean (SD)** | 15.5 (1.8) | 15.6 (2.0) | 15.4 (1.7) | .039 |
| **Gender** |  |  |  | <.001 |
| Female | 9,641 (49.1) | 3,726 (41.1) | 5,915 (54.5) |  |
| Male | 9,281 (50.9) | 4,778 (58.9) | 4,503 (45.5) |  |
| **Race/ethnicity** |  |  |  | <.001 |
| Black | 3,790 (16.3) | 1,648 (20.3) | 2,142 (14.2) |  |
| Latinx | 3,230 (13.2) | 1,484 (16.6) | 1,746 (11.4) |  |
| White | 9,608 (70.5) | 3,078 (63.1) | 6,530 (74.4) |  |
| **Highest parent education** |  |  |  | <.001 |
| Less than high school | 1,949 (10.2) | 981 (12.8) | 968 (8.5) |  |
| High school diploma | 5,410 (32.1) | 2,432 (33.9) | 2,978 (30.9) |  |
| Some college | 3,878 (21.6) | 1,647 (20.5) | 2,231 (22.3) |  |
| College degree or more | 7,051 (36.2) | 3,060 (32.9) | 3,991 (38.3) |  |
| **Household income** |  |  |  | <.001 |
| <25K | 4,202 (22.8) | 2,009 (25.3) | 2,193 (21.2) |  |
| 25K-49K | 4,649 (25.8) | 1,959 (24.9) | 2,690 (26.3) |  |
| 50K-74K | 3,177 (17.7) | 1,245 (14.9) | 1,932 (19.6) |  |
| ≥75K | 1,991 (10.6) | 748 (8.7) | 1,243 (11.9) |  |
| Missing | 4,905 (23.1) | 2,545 (26.3) | 2,360 (21.0) |  |
| **Family structure** |  |  |  | <.001 |
| Two biological parents | 9,686 (53.3) | 4,049 (48.0) | 5,637 (57.0) |  |
| Two parents (≥1 non-bio parent) | 3,444 (17.2) | 1,641 (18.7) | 1,803 (16.1) |  |
| Single parent | 4,644 (23.5) | 2,198 (25.7) | 2,446 (21.9) |  |
| Other | 1,150 (6.0) | 618 (7.6) | 532 (5.0) |  |
| **Household language** |  |  |  | <.001 |
| English | 16,734 (92.3) | 7,153 (88.9) | 9,581 (94.7) |  |
| Spanish | 1,511 (5.3) | 736 (6.1) | 775 (4.7) |  |
| Other | 669 (2.4) | 607 (5.0) | 62 (0.6) |  |
| **Self-reported health** |  |  |  | <.001 |
| Excellent | 5,300 (27.9) | 2,276 (26.7) | 3,024 (28.7) |  |
| Very good | 7,431 (39.3) | 3,234 (37.7) | 4,197 (40.4) |  |
| Good | 4,832 (25.8) | 2,291 (27.3) | 2,541 (24.8) |  |
| Fair | 1,246 (6.4) | 636 (7.6) | 610 (5.6) |  |
| Poor | 93 (0.6) | 48 (0.8) | 45 (0.5) |  |
| **Depressive symptoms, mean (SD)** | 6.6 (4.7) | 7.0 (5.1) | 6.4 (4.5) | <.001 |
| **BMI, mean (SD)** | 22.4 (4.5) | 22.3 (4.6) | 22.5 (4.3) | .023 |
| **Suicidal ideation** |  |  |  | .544 |
| Yes | 2,482 (13.2) | 1,107 (13.0) | 1,375 (13.4) |  |
| No | 16,225 (86.8) | 7,262 (87.0) | 8,963 (86.6) |  |
| Abbreviations: SD = Standard Deviation; BMI = Body Mass Index  ^a^We used an adjusted Wald F test to determine whether there were differences in means for continuous variables and a design-based F test to determine whether there were associations for categorical variables. | | | | |

| Table A2. Associations between school quality measures (Wave I) and academic outcomes (Wave IV), stratified by participant race/ethnicity, National Longitudinal Survey of Adolescent Health 1994-2008. | | | | | | |
| --- | --- | --- | --- | --- | --- | --- |
|  | **High School Graduation** | | | **College Attendance** | | |
|  | **White** | **Black** | **Latinx** | **White** | **Black** | **Latinx** |
| Student promotion rate (OR [CI]) | **1.30 [1.120, 1.520]*** | 1.21 [0.985, 1.480] | 1.00 [0.790, 1.266] | 1.08 [0.985, 1.183] | 1.07 [0.939, 1.216] | 1.06 [0.929, 1.203] |
| Teacher retention  (OR [CI]) | **1.29 [0.999, 1.677]** | 1.18 [0.746, 1.876] | 1.10 [0.769, 1.579] | 1.03 [0.932, 1.145] | 0.78 [0.599, 1.017] | 1.21 [0.939, 1.547] |
| Average Daily Attendance (OR [CI]) |  |  |  |  |  |  |
| High | ref | ref | ref | ref | ref | ref |
| Medium | 1.36 [0.820, 2.258] | 0.94 [0.422, 2.099] | 1.26 [0.483, 3.302] | 0.99 [0.815, 1.204] | 0.90 [0.580, 1.386] | 0.99 [0.633, 1.538] |
| Low | **0.40 [0.218, 0.737]*** | 0.74 [0.315, 1.747] | 0.45 [0.179, 1.151] | **0.65 [0.478, 0.892]*** | **0.59 [0.369, 0.957]*** | 1.12 [0.724, 1.739] |
| Parental involvement (OR [CI]) |  |  |  |  |  |  |
| High | ref | ref | ref | ref | ref | ref |
| Medium | 1.06 [0.555, 2.007] | **2.55 [1.047, 6.200]*** | 0.91 [0.252, 3.261] | 0.99 [0.726, 1.359] | **1.98 [1.075, 3.660]*** | 1.13 [0.664, 1.933] |
| Low | 0.70 [0.376, 1.314] | 0.57 [0.219, 1.473] | **0.35 [0.133, 0.939]*** | 0.89 [0.672, 1.186] | 0.94 [0.606, 1.466] | 1.09 [0.689, 1.720] |
| No PTA | 1.07 [0.535, 2.149] | 0.67 [0.200, 2.229] | 0.66 [0.148, 2.925] | 1.30 [0.906, 1.862] | 0.77 [0.398, 1.490] | 0.83 [0.348, 1.992] |
| Abbreviations: CI = 95% confidence interval, OR = Odds Ratio  *p<0.05  Separate models constructed for each school quality measure and academic outcome pair. All models adjusted for age, sex, household income, parental education, family structure, household language, school size, school type, school urbanicity, school region, and whether it was a middle or high school. | | | | | | |

| Table A3. Associations between school quality measures (Wave I) and health outcomes (Wave V), stratified by participant race/ethnicity, National Longitudinal Survey of Adolescent Health 1994-2008. | | | | | | |
| --- | --- | --- | --- | --- | --- | --- |
|  | **Self-Rated Health** | | | **Depressive Symptoms** | | |
|  | **White** | **Black** | **Latinx** | **White** | **Black** | **Latinx** |
| Student promotion rate (*β* [CI]) | -0.02 [-0.050, 0.011] | 0.01 [-0.021, 0.050] | 0.00 [-0.059, 0.063] | -0.05 [-0.174, 0.076] | 0.05 [-0.077, 0.167] | -0.14 [-0.316, 0.028] |
| Teacher retention  (*β* [CI]) | -0.03 [-0.061, 0.004] | **0.08 [0.000, 0.152]** | 0.07 [-0.031, 0.166] | -0.08 [-0.202, 0.046] | **0.42 [0.153, 0.690]*** | **0.23 [0.003, 0.466]*** |
| Average Daily Attendance (*β* [CI]) |  |  |  |  |  |  |
| High | ref | ref | ref | ref | ref | ref |
| Medium | 0.02 [-0.038, 0.070] | -0.02 [-0.138, 0.104] | -0.06 [-0.230, 0.103] | 0.12 [-0.049, 0.293] | -0.31 [-0.825, 0.200] | 0.36 [-0.183, 0.905] |
| Low | 0.11 [-0.019, 0.234] | -0.01 [-0.136, 0.122] | -0.03 [-0.280, 0.212] | 0.09 [-0.307, 0.479] | -0.37 [-0.891, 0.160] | **0.98 [0.258, 1.697]*** |
| Parental involvement (*β* [CI]) |  |  |  |  |  |  |
| High | ref | ref | ref | ref | ref | ref |
| Medium | 0.02 [-0.052, 0.101] | 0.19 [-0.042, 0.413] | 0.05 [-0.134, 0.232] | 0.09 [-0.184, 0.373] | **-0.59 [-1.181, -0.004]*** | -0.37 [-0.996, 0.251] |
| Low | 0.04 [-0.043, 0.113] | -0.09 [-0.243, 0.072] | 0.18 [-0.015, 0.383] | -0.04 [-0.276, 0.193] | -0.16 [-0.680, 0.368] | 0.33 [-0.212, 0.870] |
| No PTA | 0.00 [-0.092, 0.087] | -0.06 [-0.257, 0.144] | -0.31 [-0.632, 0.021] | -0.10 [-0.381, 0.177] | -0.17 [-0.921, 0.574] | -0.40 [-1.054, 0.260] |
| Abbreviations: CI = 95% confidence interval  *p<0.05  Separate models constructed for each school quality measure and health outcome pair. All models adjusted for age, sex, household income, parental education, family structure, household language, school size, school type, school urbanicity, school region, and whether it was a middle or high school. | | | | | | |

| Table A3 (continued). Associations between school quality measures (Wave I) and health outcomes (Wave V), stratified by participant race/ethnicity, National Longitudinal Survey of Adolescent Health 1994-2008. | | | | | | |
| --- | --- | --- | --- | --- | --- | --- |
|  | **Depressive Diagnosis** | | | **Suicidal Ideation** | | |
|  | **White** | **Black** | **Latinx** | **White** | **Black** | **Latinx** |
| Student promotion rate (OR [CI]) | 1.02 [0.918, 1.134] | 1.12 [1.004, 1.252] | 0.95 [0.766, 1.178] | 0.90 [0.807, 1.015] | 1.21 [0.985, 1.488] | 0.90 [0.675, 1.193] |
| Teacher retention  (OR [CI]) | 1.00 [0.917, 1.092] | 1.28 [0.944, 1.729] | 0.96 [0.655, 1.402] | 0.87 [0.747, 1.007] | **1.81 [1.384, 2.368]** | 1.36 [0.868, 2.122] |
| Average Daily Attendance (OR [CI]) |  |  |  |  |  |  |
| High | ref | Ref | ref | ref | ref | ref |
| Medium | **3.23 [2.721, 3.946]** | **0.53 [0.344, 0.828]** | 1.18 [0.534, 2.625] | 1.17 [0.893, 1.523] | 1.23 [0.589, 2.567] | 0.82 [0.281, 2.400] |
| Low | 4.13 [2.675, 7.744] | **0.58 [0.358, 0.941]** | 0.95 [0.426, 2.111] | 1.05 [0.622, 1.779] | 0.80 [0.376, 1.719] | 2.11 [0.553, 8.062] |
| Parental involvement (OR [CI]) |  |  |  |  |  |  |
| High | ref | ref | ref | ref | ref | ref |
| Medium | 1.24 [0.994, 1.551] | 0.78 [0.271, 2.277] | 1.08 [0.532, 2.178] | 0.96 [0.627, 1.457] | 0.41 [0.139, 1.187] | 1.04 [0.289, 3.743] |
| Low | 1.12 [0.885, 1.147] | 0.85 [0.561, 1.295] | 0.94 [0.464, 1.918] | 0.86 [0.606, 1.225] | 0.60 [0.315, 1.157] | 1.97 [0.698, 5.561] |
| No PTA | 1.23 [0.936, 1.629] | 1.07 [0.594, 1.934] | 0.86 [0.382, 1.958] | 1.02 [0.694, 1.493] | 1.40 [0.639, 3.052] | 1.31 [0.300, 5.709] |
| Abbreviations: CI = 95% confidence interval, OR = Odds Ratio  *p<0.05  Separate models constructed for each school quality measure and health outcome pair. All models adjusted for age, sex, household income, parental education, family structure, household language, school size, school type, school urbanicity, school region, and whether it was a middle or high school. | | | | | | |

| Table A3 (continued). Associations between school quality measures (Wave I) and health outcomes (Wave V), stratified by participant race/ethnicity, National Longitudinal Survey of Adolescent Health 1994-2008. | | | | | | |
| --- | --- | --- | --- | --- | --- | --- |
|  | **High Cholesterol Diagnosis** | | | **Hypertension Diagnosis** | | |
|  | **White** | **Black** | **Latinx** | **White** | **Black** | **Latinx** |
| Student promotion rate (OR [CI]) | 1.04 [0.936, 1.162] | 0.95 [0.843, 1.082] | 0.93 [0.764, 1.138] | 1.10 [0.973, 1.244] | 0.93 [0.846, 1.031] | 0.99 [0.846, 1.166] |
| Teacher retention  (OR [CI]) | 0.94 [0.834, 1.049] | 0.85 [0.657, 1.106] | 1.18 [0.879, 1.576] | 0.95 [0.850, 1.067] | 1.13 [0.982, 1.303] | 0.99 [0.790, 1.229] |
| Average Daily Attendance (OR [CI]) |  |  |  |  |  |  |
| High | ref | ref | ref | ref | ref | ref |
| Medium | 0.91 [0.724, 1.150] | 0.88 [0.621, 1.239] | 1.04 [0.596, 1.804] | 1.01 [0.822, 1.242] | 0.96 [0.710, 1.300] | 0.80 [0.434, 1.482] |
| Low | 1.07 [0.783, 1.469] | 0.84 [0.556, 1.259] | 1.30 [0.795, 2.117] | 0.92 [0.622, 1.354] | 0.86 [0.614, 1.211] | 0.97 [0.519, 1.830] |
| Parental involvement (OR [CI]) |  |  |  |  |  |  |
| High | ref | ref | ref | ref | ref | ref |
| Medium | 0.92 [0.702, 1.210] | 0.74 [0.413, 1.326] | 1.05 [0.555, 1.995] | 1.14 [0.888, 1.453] | 1.22 [0.807, 1.846] | 1.24 [0.618, 2.492] |
| Low | 0.80 [0.602, 1.075] | 0.99 [0.687, 1.428] | 0.87 [0.541, 1.404] | 1.08 [0.803, 1.455] | 1.25 [0.886, 1.765] | 0.74 [0.405, 1.356] |
| No PTA | 0.92 [0.675, 1.263] | 1.17 [0.688, 1.981] | 1.24 [0.482, 3.176] | 1.16 [0.882, 1.517] | 1.29 [0.743, 2.239] | 0.83 [0.383, 1.798] |
| Abbreviations: CI = 95% confidence interval, OR = Odds Ratio  *p<0.05  Separate models constructed for each school quality measure and health outcome pair. All models adjusted for age, sex, household income, parental education, family structure, household language, school size, school type, school urbanicity, school region, and whether it was a middle or high school. | | | | | | |

| Table A3 (continued). Associations between school quality measures (Wave I) and health outcomes (Wave V), stratified by participant race/ethnicity, National Longitudinal Survey of Adolescent Health 1994-2008. | | | | | | |
| --- | --- | --- | --- | --- | --- | --- |
|  | **Diabetes Mellitus Diagnosis** | | | **Cardiovascular Disease** | | |
|  | **White** | **Black** | **Latinx** | **White** | **Black** | **Latinx** |
| Student promotion rate (OR [CI]) | 0.95 [0.795, 1.147] | 1.08 [0.951, 1.237] | 1.06 [0.856, 1.304] | 1.03 [0.795, 1.322] | 0.84 [0.664, 1.064] | 0.90 [0.627, 1.284] |
| Teacher retention  (OR [CI]) | 1.07 [0.883, 1.301] | 1.17 [0.830, 1.646] | 0.98 [0.646, 1.474] | 0.93 [0.667, 1.298] | 0.86 [0.513, 1.439] | 1.33 [0.503, 3.536] |
| Average Daily Attendance (OR [CI]) |  |  |  |  |  |  |
| High | ref | ref | ref | ref | ref | ref |
| Medium | 1.03 [0.762, 1.402] | 1.04 [0.668, 1.616] | 0.97 [0.496, 1.897] | 1.74 [0.940, 3.235] | 0.63 [0.201, 1.977] | 2.09 [0.202, 21.600] |
| Low | **2.11 [1.074, 4.140]** | 0.68 [0.408, 1.130] | 0.89 [0.472, 1.687] | 2.21 [0.912, 5.340] | 1.07 [0.404, 2.818] | 4.65 [0.647, 33.435] |
| Parental involvement (OR [CI]) |  |  |  |  |  |  |
| High | ref | ref | ref | ref | ref | ref |
| Medium | 1.48 [0.850, 2.574] | 1.34 [0.763, 2.353] | 1.16 [0.466, 2.865] | 2.23 [0.989, 5.030] | 2.05 [0.590, 7.120] | 0.31 [0.098, 1.000] |
| Low | 1.30 [0.821, 2.068] | 0.96 [0.579, 1.587] | 1.35 [0.590, 3.083] | 1.22 [0.498, 3.004] | 3.07 [0.872, 10.833] | 0.22 [0.044, 1.128] |
| No PTA | 1.21 [0.719, 2.037] | 1.63 [0.788, 3.388] | 0.76 [0.103, 5.607] | 1.03 [0.459, 2.310] | 0.71 [0.097, 5.148] | - |
| Abbreviations: CI = 95% confidence interval, OR = Odds Ratio, - = insufficient subsample size.  *p<0.05  Separate models constructed for each school quality measure and health outcome pair. All models adjusted for age, sex, household income, parental education, family structure, household language, school size, school type, school urbanicity, school region, and whether it was a middle or high school. | | | | | | |

| Table A4. Associations between school quality measures (Wave I) and health outcomes (Wave V), using an ordinal logistic regression and stratified by participant race/ethnicity, National Longitudinal Survey of Adolescent Health 1994-2008. | | | | | | |
| --- | --- | --- | --- | --- | --- | --- |
|  | **Self-Rated Health** | | | **Depressive Symptoms** | | |
|  | **White** | **Black** | **Latinx** | **White** | **Black** | **Latinx** |
| Student promotion rate (OR [CI]) | 0.950 [0.890,1.014] | 1.036 [0.964,1.114] | 1.008 [0.899,1.129] | 0.968 [0.889,1.054] | 1.042 [0.963,1.126] | 0.904 [0.795,1.027] |
| Teacher retention  (OR [CI]) | 0.949 [0.890,1.012] | **1.147 [0.981,1.342]** | 1.117 [0.914,1.367] | 0.975 [0.896,1.061] | **1.209 [1.020,1.432]*** | **1.173 [0.982,1.401]** |
| Average Daily Attendance (OR [CI]) |  |  |  |  |  |  |
| High | ref | ref | ref | ref | ref | ref |
| Medium | 1.021 [0.918,1.134] | 0.962 [0.752,1.232] | 0.886 [0.625,1.254] | 1.090 [0.973,1.220] | 0.822 [0.617,1.096] | 1.160 [0.755,1.780] |
| Low | 1.244 [0.954,1.622] | 0.986 [0.763,1.274] | 0.891 [0.545,1.458] | 1.112 [0.840,1.471] | 0.787 [0.588,1.051] | **1.747 [1.087,2.807]*** |
| Parental involvement (OR [CI]) |  |  |  |  |  |  |
| High | ref | ref | ref | ref | ref | ref |
| Medium | 1.074 [0.922,1.251] | 1.480 [0.931,2.351] | 1.082 [0.741,1.580] | 1.007 [0.845,1.199] | 0.863 [0.620,1.202] | 0.752 [0.451,1.251] |
| Low | 1.083 [0.931,1.261] | 0.851 [0.614,1.179] | 1.324 [0.888,1.973] | 0.970 [0.825,1.140] | 0.928 [0.679,1.267] | 1.244 [0.776,1.993] |
| No PTA | 1.018 [0.861,1.202] | 0.919 [0.614,1.375] | 0.533 [0.267,1.063] | 0.871 [0.722,1.052] | 0.893 [0.531,1.502] | 0.856 [0.485,1.513] |
| Abbreviations: CI = 95% confidence interval  *p<0.05  Separate models constructed for each school quality measure and health outcome pair. All models adjusted for age, sex, household income, parental education, family structure, household language, school size, school type, school urbanicity, school region, and whether it was a middle or high school. | | | | | | |

### Figures

Figure A1. Study sample selection diagram

Caption

Flow diagram depicting the process of analytic sample creation, including the initial sample size from the Wave 1 in-home survey, exclusions due to loss of follow-up and specific exclusion criteria, and the final analytic sample. Excluded cases may have more than one exclusion criteria.

Figure A2. Associations between school quality measures (Wave I) and health outcomes (Wave V), stratified by participant race/ethnicity, National Longitudinal Survey of Adolescent Health 1994-2008.

A.

Caption:

Horizontal bars represent 95% confidence intervals around the estimates. Separate models were constructed for each school quality measure and health outcome pair. All models adjusted for age, sex, household income, parental education, family structure, household language, school size, school type, school urbanicity, school region, and whether it was a middle or high school. Cardiovascular disease (F) is a composite outcome of any of: heart disease, heart failure, or stroke. Statistical interactions between school quality indicators and race/ethnicity were significant in the following comparisons: (A) none; (B) White-Latinx 90-94% average daily attendance (*p =* 0.020), White-Black student promotion rate (*p =* 0.003), White-Latinx teacher retention (*p =* 0.014); (C) none; (D) Black-Hispanic student promotion rate (*p =* 0.023) and White-Black teacher retention (*p =* 0.021); (E) none; (F) none.

### Post-hoc Analyses

We performed select exploratory analyses to better understand our main findings. In two separate sets of models, we added *college attendance* and, separately, *having reported less respect because of race* as mediators of the association between each school quality measure and each outcome (see Appendix above for description of post-hoc data analyses). The findings were similar to our primary analyses and did not uncover a convincing pattern of statistically significant mediation in our models.

| Table A4. Associations between school quality measures (Wave I) and self-reported health and depressive symptoms (Wave V) as mediated by college attendance (Wave III) and experiencing less respect due to race (Wave IV), stratified by participant race/ethnicity, National Longitudinal Survey of Adolescent Health 1994-2008. | | | | | | |
| --- | --- | --- | --- | --- | --- | --- |
|  | **Self-Reported Health** | | | | | |
|  | **Less Respect Due to Race** | | | **College Attendance** | | |
|  | **White** | **Black** | **Latinx** | **White** | **Black** | **Latinx** |
| Student promotion rate (*β* [CI]) | -0.03 [-0.064, 0.008] | 0.02 [-0.020, 0.063] | -0.02 [-0.089, 0.044] | -0.01 [-0.044, 0.017] | 0.02 [-0.016, 0.052] | 0.01 [-0.058, 0.069] |
| Teacher retention  (*β* [CI]) | -0.03 [-0.067, 0.013] | 0.08 [-0.012, 0.168] | 0.05 [-0.047, 0.155] | -0.03 [-0.060, 0.004] | 0.07 [-0.010, 0.143] | 0.08 [-0.023, 0.186] |
| Average Daily Attendance (*β* [CI]) |  |  |  |  |  |  |
| High | Ref | Ref | Ref | Ref | Ref | Ref |
| Medium | 0.04 [-0.027, 0.103] | -0.05 [-0.175, 0.081] | -0.06 [-0.248, 0.136] | 0.02 [-0.035, 0.070] | -0.02 [-0.145, 0.098] | -0.07 [-0.243, 0.112] |
| Low | **0.15 [0.025, 0.280]** | -0.01 [-0.148, 0.135] | 0.07 [-0.208, 0.350] | 0.07 [-0.045, 0.190] | -0.01 [-0.143, 0.116] | -0.02 [-0.280, 0.231] |
| Parental involvement (*β* [CI]) |  |  |  |  |  |  |
| High | Ref | Ref | Ref | Ref | Ref | Ref |
| Medium | 0.01 [-0.072, 0.096] | 0.22 [-0.010, 0.453] | 0.11 [-0.073, 0.298] | 0.02 [-0.049, 0.097] | 0.20 [-0.019, 0.423] | 0.06 [-0.130, 0.243] |
| Low | 0.01 [-0.081, 0.102] | 0.00 [-0.148, 0.155] | **0.19 [0.015, 0.358]*** | 0.03 [-0.048, 0.099] | -0.08 [-0.234, 0.066] | 0.19 [-0.022, 0.403] |
| No PTA | 0.01 [-0.090, 0.107] | -0.00 [-0.200, 0.199] | -0.20 [-0.582, 0.172] | 0.01 [-0.073, 0.095] | -0.05 [-0.235, 0.136] | -0.32 [-0.674, 0.032] |
| Abbreviations: CI = 95% confidence interval, OR = Odds Ratio  *p<0.05  Separate models constructed for each school quality measure and academic outcome pair. All models adjusted for age, sex, household income, parental education, family structure, household language, school size, school type, school urbanicity, school region, and whether it was a middle or high school. | | | | | | |

| Table A4 continued. Associations between school quality measures (Wave I) and self-reported health and depressive symptoms (Wave V) as mediated by college attendance (Wave III) and experiencing less respect due to race (Wave IV), stratified by participant race/ethnicity, National Longitudinal Survey of Adolescent Health 1994-2008. | | | | | | |
| --- | --- | --- | --- | --- | --- | --- |
|  | **Depressive Symptoms** | | | | | |
|  | **Less Respect Due to Race** | | | **College Attendance** | | |
|  | **White** | **Black** | **Latinx** | **White** | **Black** | **Latinx** |
| Student promotion rate (*β* [CI]) | -0.05 [-0.179, 0.084] | 0.01 [-0.108, 0.134] | -0.09 [-0.296, 0.115] | -0.04 [-0.165, 0.088] | 0.05 [-0.070, 0.172] | -0.14 [-0.313, 0.031] |
| Teacher retention  (*β* [CI]) | -0.02 [-0.136, 0.102] | 0.27 [-0.064, 0.596] | 0.22 [-0.008, 0.448] | -0.08 [-0.203, 0.045] | **0.40 [0.135, 0.672]*** | **0.25 [0.006, 0.489]*** |
| Average Daily Attendance (*β* [CI]) |  |  |  |  |  |  |
| High | Ref | Ref | Ref | Ref | Ref | Ref |
| Medium | 0.17 [-0.003, 0.351] | -0.06 [-0.658, 0.539] | 0.17 [-0.406, 0.737] | 0.12 [-0.048, 0.296] | -0.33 [-0.840, 0.182] | 0.36 [-0.187, 0.908] |
| Low | 0.08 [-0.343, 0.511] | -0.06 [-0.602, 0.476] | 0.65 [-0.043, 1.340] | 0.01 [-0.390, 0.411] | -0.39 [-0.898, 0.127] | **1.00 [0.282, 1.712]*** |
| Parental involvement (*β* [CI]) |  |  |  |  |  |  |
| High | Ref | Ref | Ref | Ref | Ref | Ref |
| Medium | 0.07 [-0.234, 0.383] | **-0.60 [-1.209, -0.000]*** | -0.26 [-0.816, 0.300] | 0.10 [-0.183, 0.374] | **-0.58 [-1.151, -0.015]*** | -0.37 [-0.991, 0.254] |
| Low | -0.03 [-0.279, 0.215] | 0.06 [-0.448, 0.566] | 0.53 [-0.033, 1.099] | -0.06 [-0.294, 0.173] | -0.15 [-0.682, 0.372] | 0.33 [-0.222, 0.878] |
| No PTA | -0.18 [-0.482, 0.122] | 0.01 [-0.694, 0.709] | -0.15 [-0.752, 0.444] | -0.09 [-0.369, 0.196] | -0.18 [-0.932, 0.566] | -0.41 [-1.078, 0.250] |
| Abbreviations: CI = 95% confidence interval, OR = Odds Ratio  *p<0.05  Separate models constructed for each school quality measure and academic outcome pair. All models adjusted for age, sex, household income, parental education, family structure, household language, school size, school type, school urbanicity, school region, and whether it was a middle or high school. | | | | | | |
